# Triatomine bugs (Hemiptera, Reduviidae, Triatominae) in the Domiciles of the Guaribas Valley Territory, in Northeastern Brazil

**DOI:** 10.1590/0037-8682-0177-2020

**Published:** 2020-09-11

**Authors:** Antonio Ferreira Mendes-Sousa, Leid Daiane Neri de Araújo, Samires Silva de Sousa, Suzane de Carvalho Alencar, Wesesller Almeida de Sousa, Larisse Maria de Sousa, Suzane Maria da Rocha, José Cleves da Silva Maia, Márcia Maria Mendes Marques, Tamaris Gimenez Pinheiro, Edson Lourenço da Silva, Veruska Cavalcanti Barros, Ana Carolina Landim Pacheco

**Affiliations:** 1 Universidade Federal do Piauí, Laboratório de Parasitologia, Ecologia e Doenças Negligenciadas, Picos, PI, Brasil.; 2 Instituto Federal do Piauí, Picos, PI, Brasil.; 3 Universidade Federal do Piauí, Centro de Inteligência de Agravos Tropicais Emergentes e Negligenciados, Teresina, PI, Brasil.

**Keywords:** Triatomine, Guaribas Valley, Brazil

## Abstract

**INTRODUCTION:**

Triatomine bugs are hematophagous insects that are extremely important in public health because they are natural vectors of *Trypanosoma cruzi*, the causative agent of Chagas disease. In this study, we aimed to assess the occurrence of triatomine species and the natural *T. cruzi* infection in the Guaribas Valley territory, an endemic region for Chagas disease in northeastern Brazil.

**METHODS:**

Insects were actively captured from July 2017 to October 2019 in the intra- and peridomiciles of 16 municipalities of the Guaribas Valley territory, in the southeast area of Piauí state. Triatomine species were identified following a taxonomic key, and natural infection was investigated through insects’ fresh feces exams.

**RESULTS:**

A total of 430 triatomines were collected, including 211 nymphs and 219 adults. Of all collected specimens, 39 (9.1%) were from the intradomiciles and 391 (90.9%) from peridomiciles. Nine species, including two subspecies, could be identified: *Triatoma brasiliensis brasiliensis, T. brasiliensis macromelasoma, T. pseudomaculata, T. sordida, T. juazeirensis, T. melanocephala, Panstrongylus lutzi, Rhodnius domesticus, R. nasutus*, and *R. robustus*. *T. brasiliensis* were the most frequently collected bugs, representing 72% of all the identified insects. None of the examined invertebrates presented flagellate forms of *T. cruzi*.

**CONCLUSIONS:**

This is the first report of *T. b. macromelasoma* and *T. juazeirensis* in the Guaribas Valley territory. The persistence of triatomine species in the domiciles in an endemic area for Chagas disease emphasizes the relevance of entomological surveillance and vector control measures in the studied area.

## INTRODUCTION

Chagas disease (CD), also known as American trypanosomiasis, is a potentially life-threatening parasitic illness caused by the protozoan parasite *Trypanosoma cruzi* (Chagas, 1909) and naturally transmitted by the feces of infected triatomine bugs (Hemiptera, Reduviidae, Triatominae). Although other types of transmissions, such as oral transmission, have gained significant attention, vectorial CD remains a public health problem in endemic areas^1^. An estimated 6-7 million people are infected with *T. cruzi*, especially in poor and disadvantaged areas in Latin America, where it remains an infection with remarkable medical and social impact and is regarded as a neglected tropical disease^2^.

In Brazil, although the number of new cases of CD has dramatically reduced in recent years, approximately 1.2-4.6 million people are infected with *T. cruzi,* causing approximately 6,000 deaths annually, making it the most lethal neglected tropical disease in the country^3^ ^,^ ^4^. The highest prevalence rates occur in the northeastern and southeastern regions of the country, presenting an estimated mean of 5.0% of prevalence in the last decades^3^. In Piauí state in the northeast region of Brazil, although the existence of CD has been suggested since 1916, the first autochthonous case was only confirmed in 1975^5^. The state is among the poorest in Brazil, 34% of its population lives in rural areas, where CD predominates^6^. Although previous studies reported decreasing seroprevalence (4% to 1.9%) in the last decades in the state^7^, the *T. cruzi* transmission still occurs, perhaps due to recent triatomine infestations. The health regions of Oeiras (5.8%), São João do Piauí (5.3%), and Picos (4.3%), which are all in the central-southern region of the state, present the highest prevalence rates for CD^6^ ^,^ ^7^.

Control measures focused on vector elimination inside houses have been successful in the country, including in the state of Piauí, and in 2006, Brazil was certified by the Pan American Health Organization/World Health Organization (PAHO/WHO) as free of CD transmission by its main vector, *Triatoma infestans* (Klug, 1834)^8^. However, native triatomine species, such as *Triatoma brasiliensis* (Neiva, 1911)*, T. pseudomaculata* (Corrêa & Espinola, 1964)*, T. sordida* (Stål, 1859) and *Panstrongylus lutzi* (Neiva & Pinto, 1923), are endemic in Piauí and present high risk of colonization inside houses besides a variable natural infection index ranging from 0.01 to 0.57 in the state^9^, increasing the possibility of vector transmission of *T. cruzi*, especially in rural areas or houses close to wild environments, where entomological surveys and control programs may be deficient^9^ ^,^ ^10^.

Knowledge of the geographical distribution and natural infections of triatomine bugs is fundamental for understanding the epidemiological aspects related to *T. cruzi* transmission and should be considered for guiding the control actions and surveillance of CD. Thus, the aim of the present work was to analyze the geographical distribution of triatomines and their natural *T. cruzi* infection in the Guaribas Valley territory, a region with endemic CD, in northeastern Brazil.

## METHODS

**FIGURE 1:**
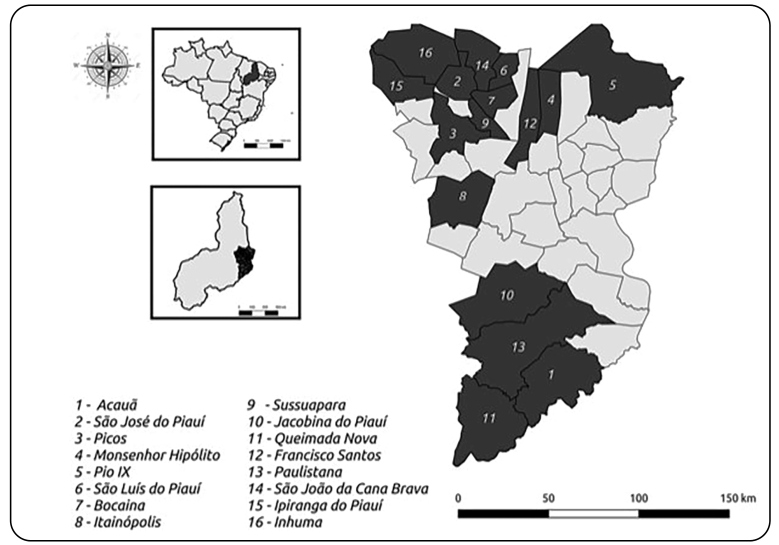
Municipalities of the Guaribas Valley territory, Piauí, Brazil, where triatomine bugs were collected.

The study was conducted in the Guaribas Valley territory in the semiarid region in the eastern part of Piauí state-one of the driest areas of Brazil, covering an area of 22,693.41 km^2^ ^11^. The Valley is composed of 39 municipalities distributed along the Guaribas River, with a population of 340,229 inhabitants, of which 180,797 (53.14%) live in rural areas^11^ and usually have poor quality human dwellings, which are favorable shelters for triatomines^9^. For this study, the triatomine bug collection occurred in houses of the municipalities of Acauã (16 houses), Bocaina (7 houses), Francisco Santos (1 house), Inhuma (2 houses), Ipiranga do Piauí (1 house), Itainópolis (13 houses), Jacobina do Piauí (1 house), Monsenhor Hipólito (3 houses), Paulistana (12 houses), Picos (7 houses), Pio IX (1 house), Queimada Nova (2 houses), São João da Canabrava (1 house), São José do Piauí (2 houses), São Luís do Piauí (3 houses), and Sussuapara (2 houses) (Figure 1).

The insects (nymphs and adults) were manually captured in the studied municipalities from July 2017 to October 2019. The collections occurred in the intra- and peridomiciles of houses where members of the community had previously reported suspicious bugs. The materials used for triatomine collection were tweezers, flashlights (to inspect cracks and sites deprived of light), and plastic containers to safe keep and transport the specimens. No dislodging substance was used in the collections. The collected material was then taken to the Laboratory of Parasitology, Ecology and Neglected Diseases of the Universidade Federal do Piauí, Picos, for gender classification, species identification^12^ ^,^ ^13^ ^,^ ^14^, and investigation of the *T. cruzi* infection. The nymphs were not taxonomically identified, as only a small proportion of the known species had morphologically described immature forms, which hampered the construction of specific taxonomic keys that allowed the identification of immature forms of all known species of triatomine bugs^13^.

Research on the flagellate forms of *T. cruzi* in the digestive tracts of living insects was performed through abdominal compression with tweezers and examination of fresh feces on glass slides. The material was then observed using an optical microscope at 40x and 100x magnification.

## RESULTS

From July 2017 to October 2019, 430 triatomine bugs were collected, including 219 adults and 211 nymphs. The insects were classified and included in 3 genera, 9 species, and 2 subspecies, as observed in Table 1. Of the 219 collected adult insects, 123 (56.2%) were female and 96 (43.8%) were male (Table 1). A total of 391 (90.9%) specimens were collected in the peridomicile, including all the nymphs (Table 1). Thirty nine specimens were collected in the intradomicile, all adult forms of 5 triatomine species and 2 subspecies (Table 1).

**TABLE 1:** Triatomine bugs collected in the Guaribas Valley territory from July 2017 to October 2019 by species, gender, and environments.

Species	Total collected	Nymphs	Adults		Intradomicile	Peridomicile
			Male	Female		
*Triatoma brasiliensis brasiliensis*	98	-	43	55	24	74
*Triatoma brasiliensis macromelasoma*	60	-	26	34	2	58
*Triatoma pseudomaculata*	27	-	12	15	5	22
*Triatoma sordida*	13	-	5	8	-	13
*Triatoma juazeirensis*	1	-	-	1	1	-
*Triatoma melanocephala*	1	-	-	1	-	1
*Triatoma* sp*.*	4	-	1	3	-	4
*Panstrongylus lutzi*	10	-	6	4	4	6
*Rhodnius domesticus*	3	-	3	-	3	-
*Rhodnius nasutus*	1	-	-	1	-	1
*Rhodnius robustus*	1	-	-	1	-	1
Not identified	211	211	-	-	-	211
**Total**	**430**	**211**	**96**	**123**	**39**	**391**

Nine triatomine species were morphologically identified. The species *T. brasiliensis* was the most prevalent, with 158 collected specimens, including 98 (62.0%) individuals belonging to the subspecies *T. b. brasiliensis* (Neiva, 1911) and 60 (38.0%) individuals of the subspecies *T. b. macromelasoma* (Galvão, 1956). *Triatoma pseudomaculata, T. sordida,* and *Panstrongylus lutzi* were the next most prevalent species, with 27, 13, and 10 collected individuals, respectively. Three samples of *Rhodnius domesticus* (Neiva & Pinto, 1923) and one each of *T. juazeirensis* (Costa & Felix, 2007)*, T. melanocephala* (Neiva & Pinto, 1923)*, R. nasutus* (Stål, 1859), and *R. robustus* (Larrousse, 1927) were also collected. Four adult invertebrates (one male and three females) were identified only as *Triatoma* sp*.*, as they did not morphologically match any of the described species (Table 1).

The highest number of triatomines (adults and nymphs) was collected in the municipality of Acauã (166 specimens), followed by Itainópolis (114 specimens), Paulistana (43 specimens), Bocaina (36 specimens), Queimada Nova (23 specimens), and Picos (22 specimens). Bocaina and Itainópolis presented the greatest varieties of triatomines, with 6 and 5 different species, respectively. The species *T. brasiliensis* was found in all municipalities except Monsenhor Hipólito (Table 2). A total of 104 collected adults triatomines were examined for the *T. cruzi* infection, but none of the examined bugs tested positive for the flagellate forms of the parasite. 

**TABLE 2:** Triatomine bugs collected in the Guaribas Valley territory from July 2017 to October 2019 by municipality.

Species	Municipality															
	Acauã	Bocaina	Francisco Santos	Inhuma	Ipiranga do Piauí	Itainópolis	Jacobina	Monsenhor Hipólito	Paulistana	Picos	Pio IX	Queimada Nova	São João da Canabrava	São José do Piauí	São Luís do Piauí	Sussuapara
*T. b. brasiliensis*	3	6	1	2	1	67	1	-	1	7	1	-	1	3	2	2
*T. b. macromelasoma*	41	-	-	-	-	-	-	-	14	-	-	5	-	-	-	-
*T. juazeirensis*	-	1	-	-	-	-	-	-	-	-	-	-	-	-	-	-
*T. melanocephala*	-	1	-	-	-	-	-	-	-	-	-	-	-	-	-	-
*T. pseudomaculata*	11	4	-	-	-	1	-	4	5	1	-	-	-	-	1	-
*T. sordida*	12	-	-	-	-	-	-	-	1	-	-	-	-	-	-	-
*Triatoma* sp*.*	4	-	-	-	-	-	-	-	-	-	-	-	-	-	-	-
*P. lutzi*	-	4	1	-	-	3	-	-	-	2	-	-	-	-	-	-
*R. domesticus*	-	-	-	1	-	1	-	-	-	1	-	-	-	-	-	-
*R. nasutus*	-	-	-	-	-	1	-	-	-	-	-	-	-	-	-	-
*R. robustus*	-	1	-	-	-	-	-	-	-	-	-	-	-	-	-	-
Nymphs	95	19	-	-	-	41	-	-	22	11	-	18	-	-	5	-
**Total**	**166**	**36**	2	3	1	**114**	1	4	**43**	**22**	1	**23**	1	3	8	2

## DISCUSSION

CD is endemic in Piauí, especially in the southeastern part of the state, including the Guaribas Valley territory^3^ ^,^ ^6^. Once the *T. cruzi* transmission by *T. infestans* was eliminated in Piauí, secondary vectors gained importance in the epidemiology of the disease due to the possibility of house colonization and re-emergence of vector-borne transmission. In the present study, triatomine bugs were found mainly in the peridomiciles, however, 39 adults were found in the intradomiciles. Several triatomine species are native to the semiarid region^9^, and CD control measures, such as domestic insecticide spraying, have been discontinued in the study area, which may have favored our findings of such a high number of bugs. 

*Triatoma brasiliensis*, the most common species, was present in almost all studied municipalities of the Guaribas Valley. Moreover, it represented the majority of the specimens collected in the intradomiciles. These findings confirmed previous studies that demonstrated *T. brasiliensis* as the most prevalent triatomine in Piauí, presenting the highest infestation rate in the state^9^ ^,^ ^15^. This species, which is commonly found in northeastern Brazil, is currently considered the most important *T. cruzi* vector in the country’s semiarid zones^15^.The control of *T. brasiliensis* remains a challenge in the northeast, as it is native to the region, and able to disperse by flight guided by artificial lights, favoring the invasion and colonization of houses even after a few months of insecticide treatment in the intradomicile^16^ ^,^ ^17^. In the present study, no specimens of *T. brasiliensis* tested positive for *T. cruzi*, confirming the tendency of decreasing natural infection of this species^15^. Our study also reports-for the first time-on the subspecies *T. brasiliensis macromelasoma* in the Guaribas Valley territory. *Triatoma b. macromelasoma* belongs to the *T. brasiliensis* complex and was restricted to the state of Pernambuco^15^. Interestingly, specimens of *T. b. macromelasoma* were collected in the municipalities of Acauã, Queimada Nova, and Paulistana, which form a frontier with Pernambuco (Figure 1). 

The species *T. pseudomaculata* was the second-most collected in the Guaribas Valley territory, found in seven of the 16 studied municipalities, predominantly in the peridomiciles. This species is commonly reported in entomological surveys in the northeast region of Brazil^18^ ^,^ ^19^, including in the state of Piauí^9^. Although usually found mainly in the peridomicile^9^ ^,^ ^19^ and described as feeding on birds^20^, recent investigations have reported *T. pseudomaculata* inside houses and feeding on human blood in central Brazil, indicating the potential of this species for intradomicile colonization and *T. cruzi* transmission^21^.

The species *P. lutzi* and *T. sordida*, which have both been previously reported in the state of Piauí^9^, were also collected in the study area. *Panstrongylus lutzi* is frequently found in the northeastern region of Brazil^18^ ^,^ ^19^ ^,^ ^22^and presents greater importance as a *T. cruzi* vector because it can be found in the intradomicile and present high levels of natural infections^22^ ^,^ ^23^. In contrast, *T. sordida* is typical of the cerrado biome (central region of Brazil) and occurs in the northeast region mainly in areas bordering that biome with the caatinga^14^. This species is predominantly observed in peridomiciles especially around bird nests and chicken coops, presenting low levels of natural infection since avians are refractory to *T. cruzi* infection^24^. 

In our study, the species *T. melanocephala*, *T. juazeirensis*, *R. domesticus*, *R. nasutus,* and *R. robustus* were found in small numbers. *Triatoma melanocephala* has been previously found in small numbers in Piauí^9^. There is a lack of information about its biological habits and role in *T. cruzi* transmission in humans, although high natural infection rates have been reported, suggesting a role in the maintenance of the sylvatic cycle of *T. cruzi* ^22^. To our knowledge, this is the first report on *T. juazeirensis* in Piauí. *Triatoma juazeirensis* belongs to the *T. brasiliensis* species complex and was first described in Bahia state, where it lives among rocks in the sylvatic environment and in the peridomicile, with potential for domiciliary infestation^25^. The species *R. domesticus* has already been reported in Piauí^26^although it predominates in areas of Atlantic forest from the southern states to Bahia in the northeast, with reports of intradomicile invasion^27^. *Rhodnius nasutus* was already found in small numbers mainly in the north of Piauí, where it presents considerably high intradomicile colonization rates^9^. *Rhodnius robustus*, despite having no colonization rate, has been dispersed throughout the state, although it was first restricted to the extreme north of Piauí^9^and currently can be found in the Guaribas Valley territory.

Firstly reported in small amounts in the southeast of Piauí in 1980^28^, the species *Triatoma infestans* has neither been found in following triatomine surveys in the state^9^, nor in our collections. The lack of occurrence of *T. infestans* in the Guaribas Valley reflected the success of the Southern Cone Initiative for elimination of the most important *T. cruzi* vector in Brazil, evidencing the interruption of CD transmission by this vector in the Piauí as in other Brazilian states^29^. In this work, none of the examined triatomine bugs presented the *T. cruzi* infection. The absence of flagellate forms in the collected bugs followed the tendency of decreasing natural infection rates in triatomines from Piauí^9^. However, investigation of natural infection by abdominal compression and examination of fecal material with optical microscopy used in this study may present low sensitivity and was performed only on a quarter of the collected bugs. With a higher number of collected specimens, the polymerase chain reaction method could improve the detection of the *T. cruzi* natural infection^30^. 

In the present study, only adult triatomine bugs were found in the intradomicile, indicating infestation but not colonization of the investigated houses. However, a high number of immature forms and adults were present in the peridomiciles, (for example, animal shelters), demonstrating colonization of these environments by triatomine species, which could be in contact with humans and synanthropic reservoirs, representing a risk of the *T. cruzi* transmission for the population of the Guaribas Valley. In this scenario, our results contribute to a better comprehension of the distribution of triatomines in the northeast region of Brazil and highlighted the importance of conducting periodic entomological surveys and preventive vector control measures against this life-threatening disease.
